# Incorporation of Buckwheat Flour at Different Particle Sizes and Distinctive Doses in Wheat Flour to Manufacture an Improved Wheat Bread

**DOI:** 10.3390/foods12081730

**Published:** 2023-04-21

**Authors:** Ionica Coţovanu, Costel Mironeasa, Silvia Mironeasa

**Affiliations:** 1 Faculty of Food Engineering, Stefan cel Mare University of Suceava, 13 Universitatii Street, 720229 Suceava, Romania; ionica.cotovanu@usm.ro; 2 Faculty of Mechanical Engineering, Automotive and Robotics, Stefan cel Mare University of Suceava, 13 Universitatii Street, 720229 Suceava, Romania

**Keywords:** optimal composite wheat–buckwheat bread, chemical composition, amino acids, minerals, rheological properties, sensory profile

## Abstract

This study explored the effect of substituting wheat flour (WF) with distinctive optimal doses of buckwheat flour (BF) corresponding to large, medium, and small particle sizes (PS), previously established based on an optimization process, on composite flour characteristics, dough rheology, and bread quality. The optimal dose for each PS was established in a previous study. The highest value for protein, lipid, mineral, and amino acids was found in the optimal composite flour with medium PS, with significant differences between those with large and small PS. The addition of BF in WF at doses corresponding to each fraction provides optimum rheological properties, with the large and medium PS providing higher performance compared to the small one. The same tendency was observed for volume and texture parameters of bread made from optimal composite flours with medium and large PS, respectively, but the crust and crumb lightness presented lower values than bread with small PS. Regarding the bread nutritional profile, the sample with medium PS possessed the highest protein, lipid, and ash content. Compared to the wheat bread, a considerably higher amino acid content, up to 21.22%, was found in bread made from optimal composite flours with medium and small PS, respectively. The bread samples with medium and large PS, respectively were superior in minerals, the value being up to 2.63 times higher compared to the control. Sensory characteristics results revealed that the bread samples containing 9.13% large and 10.57% medium PS were the most preferred by panelists. The results of this research make an important base to suitably develop wheat–buckwheat bread applications in the future.

## 1. Introduction

In general, bakery products made with refined wheat flour present a deficit of essential amino acids and minerals and must be supplemented to acquire daily physiological demands [1,2]. Supplementing refined flour with other flours with higher nutritional quality than wheat flour is a frequent practice [3,4,5]. The addition of buckwheat flour in bread formulation has an essential nutritional impact but its successful implementation is challenging and requires a good understanding of the effect of adequate flour granulometry and substitution level on bread quality. The replacement of buckwheat flour in wheat flour mixtures represents an alternative for developing cereal-based products with increased added value [6,7,8]. The quality attributes of cereal-based products from the point of view of added nutritional value, taste, convenience, and easy manipulation during processing support pseudocereals as suitable for obtaining a highly nutritious and innovative baked product [2,9,10].

Previous research revealed that the incorporation of whole pseudocereal flour, especially in large amounts, decreased yield, volume, fermentation parameters, crumb elasticity, and modified the flavor, depending on pseudocereals and type of bread, because of the gluten matrix dilution [11,12,13]. Many researchers presented the use of high nutritional pseudocereals in bakery product manufacturing and concluded that it would be necessary to make changes in the traditional bread-making process. In this case, it could include higher doses of buckwheat flour in bread for obtaining new bread varieties with high nutritional and sensory value [14].

The partial replacement of wheat flour with buckwheat flour in bread has been studied by different groups of researchers [15,16,17,18]. It has been stated that, buckwheat can be added into bread to obtain a product with more carbohydrates, amino acids, strong umami palatability, and a characteristic flavor [19]. Yildiz and Bilgiçli [20,21] searched the possibility of adding buckwheat flour, up to 30% in gluten-free flatbread (lavas) and more than 40% in gluten-free bread, which raised the nutritional quality, except the phytate content. The darker color and the bitter taste negatively influenced the scoring scale given to the bread with a 40% buckwheat flour substitution [20]. Buckwheat has also been shown to have the power to help develop products with a reduced glycemic index [22]. The amount of resistant starch in bread products with different additions (30–70%) of buckwheat flour increased (from 0.9% to 4.4%) compared to that of the control sample (0.8%), and the in vitro amylolysis digestion rate was substantially lower compared to that for wheat flour bread [22].

A bread sensory properties evaluation demonstrated that buckwheat flour incorporation in wheat flour can potentiate the quality attributes of overall acceptability, specifically taste, color, and smell [23].

The influence of yeast in the preparation of buckwheat bread was researched by Moroni et al. [24,25]. Extensive hydrolysis of the globulin fraction and release of small polypeptides occurred after fermentation, and malt-containing buckwheat induced inhibition of CO_2_ production by baker’s yeast during fermentation [24]. However, the features of wheat flour bread were improved by adding 10% buckwheat sourdough, which led to a higher specific volume and a softer crumb. The fermentation process positively influenced the nutritional properties from the point of view of the content of polyphenols and phytates [25]. Among cereals, only wheat has the ability to form a strong and cohesive dough, able to retain gases and provide a spongy product for baking [26]. Gluten is the wheat protein that is recognized to be responsible for the unique viscoelastic texture of wheat flour dough [26]. Pseudocereal seed proteins are not able to form dough because they do not contain gluten. Therefore, pseudocereal flour is used in mixtures with wheat flour and other cereals to obtain bakery products, but the dosage of addition in flour products is limited [27,28,29]. Besides the addition of the dose, another factor that influences the quality parameters of the finished product is the particle size of the pseudocereal flour. Kurek et al. [30] affirmed that replacing quinoa flour with different particle sizes significantly impacted the chemical characteristics of bread and the consumer’s acceptability. Sciarini et al. [13] stated that the finest fractions of buckwheat and quinoa resulted in breads with higher technological quality, as well as a final product with more fiber, minerals, and proteins. In another study on a non-gluten flour, it was reported that the particle size of corn flour affected the dough development of gluten-free bread during fermentation and, therefore, the final volume and texture of the breads [31].

In our previous study [16], we investigated the effect of substituting wheat flour with buckwheat flour and its fractions (large, medium, and small) at different levels (0, 5, 10, 15, and 20%) on wheat flour amylase activity, dough behavior during mixing, heating–cooling stages, biaxial extension, rheofermentation, oscillatory and creep-recovery tests, and bread quality parameters. The following parameters were assessed: Falling Number index (FN); Mixolab parameters—water absorption (WA), dough development time (DT), dough stability (ST), speed of protein weakening (C1-2), starch gelatiniyation (C3-2), cooking stability (C3-4), and starch retrogradation (C5-4); alveograph parameters—dough elasticity (P), dough extensibility (L), deformation energy (W), and P/L alveograph ratio, Rheofermentomenters parameters—maximum height of the gas release curve (H′_m_), total volume of gas produced (TV), volume of the gas retained in the dough (VR), and retention coefficient (CR); rheometer parameters—storage modulus (G′), viscous modulus (G″), loss tangent (tg δ), maximum gelatinization temperature (Tmax), maximum creep-recovery compliance (Jcmax, Jrmax); as well as bread volume (BV), and bread hardness (BH). Additionally, the optimal wheat–buckwheat composite flour coresponding to each fraction was established by applying multiple response analysis to the predictive models generated for empirical and dynamic rheological properties of the dough, as well as on some of the technical characteristics of the bread, in conjunction with the desirability function approach. For this purpose, by imposing the following desired goals for responses: ST, C3-4, H′_m_, TV, VR, CR, W, and J_rmax_ set at maximum value; minimization of C1-2, C5-4, P/L; and the level of all remaining responses maintained within range, the maximum dose possibility for each fraction which can substitute wheat flour and give the best technological characteristics for composite flour and bread quality was established.

This paper continues our previous work and presents a complete characterization of the optimal products obtained in there. To our knowledge, there is a scarcity of research about the influence of the optimal wheat–buckwheat composite flour containing large, medium, and small buckwheat flour particle sizes on the physical properties, sensorial perception, and nutritional profile of the obtained bread. Therefore, the aim of this study was to make a complete characterization from a technological and a nutritional point of view of optimal wheat–buckwheat composite flours with large, medium, and small particle sizes, as established in our previously study. In addition, the bread features were evaluated. This work could contribute to the design of high-quality flour-based products enriched with different buckwheat particle sizes in the future.

## 2. Materials and Methods

### 2.1. Raw Materials

The wheat flour (fat 1.40%; protein 14.00%, ash 0.65%; wet gluten content 30.00%; Falling Number index 312 s; gluten deformation index 6.00 mm) was supplied by a bread factory (Mopan, Suceava, România), the fresh *Saccharomyces cerevisiae* yeast was purchased from Rompak company (Paşcani, România), and the salt and buckwheat seeds were bought at a market (SanoVita, Vâlcea, România). The buckwheat composition consisted of total protein (13.26%), moisture (13.28%), fat (3.40%), and ash (2.00%) on a dry weight basis. Buckwheat seeds with coatings were milled and sieved according to our previous research [16,32], to obtain three particle sizes (PS): large (L > 300 μm), medium (M > 180 μm, ˂300 μm), and small fractions (S < 180 μm).

### 2.2. Preparation of Optimal Composite Flour

In our previous research [16] we determined the optimal proportion wheat:buckwheat flour for each buckwheat particle size (PS). The proportions were 90.87:9.13% for large (OF_BL), 89.43:10.57% for medium (OF_BM), and 89.75:10.25% for small (OF_BS) particle size. These amounts of wheat flour and buckwheat flour were mixed in a Yucebas Y21 machine (Izmir, Turkey) for a half hour to formulate optimal composite flours. Wheat flour (WF) without buckwheat was considered as control.

### 2.3. Dough and Bread-Making Procedure

Breads were made through the biphasic dough method, as previously described by Cotovanu et al. [6] from WF (control) or optimal composite flours. The amounts of raw materials were: 300 g flour, 9 g yeast, 0.54 g salt, and water. The required amount of water has been established at Mixolab (Chopin, Tripetteet Renaud, Paris, France) [33]. For dynamic, empirical, and texture determination, the dough was prepared only with flour and water. The biphasic method consists of manually mixing the yeast with water and a moiety of flour, and the mixture is then kept in a fermentation chamber (30 °C and 85% RH) for 2 h (PL2008, Piron, Cadoneghe, Padova, Italy). After fermentation, the other part of the flour and salt are added and mixing continues for 10 min with a mixer (Kitchen Aid, Whirlpool Corporation, Benton Harbor, MI, USA). The dough is left in the fermentation room for 1 h, and then it is cut into pieces of 400 g and placed in trays to finish the fermentation for another hour, and finally baked in a Caboto PF8004D (Cadoneghe, Padova, Italy) at 200 °C, for half hour.

### 2.4. Proximate Analysis of Samples

The moisture, ash, protein, and lipids, expressed as % (dry basis), were determined according to the International Association for Cereal Science and Technology (ICC) methods [34]. The moisture content of flours and breads was determined by oven-drying (Zhicheng Analysis Instruments, Shanghai, China) at 103 °C until constant weight, based on the gravimetric method (ICC 110/1). Ash content was determined according to the ICC 104/1 by using a muffle furnace (Nabertherm, LE 2/11/R6, Bremen, Germany) until the material became white. The protein content was determined according to the ICC 105/2 method by using the Kjeldahl method with digestion and steam distillation (VELP Scientifica, Usmate Velate (MB), Italy). Lipids content was determined according to the ICC 136 using an automatic Soxhlet extraction system (VELP Scientifica, Usmate Velate (MB), Italy) with n-hexane solvent. The carbohydrate content was estimated by difference, while the breads’ energetic value was calculated following the method mentioned by Raczyk et al. [35].

### 2.5. Minerals Content Determination

The mineral contents, phosphorus (P), zinc (Zn), iron (Fe), calcium (Ca), magnesium (Mg), potassium (K), and sodium (Na) of the flour and bread samples were determined by using a flame atomic absorption spectrometry (FAAS) (AA-6300 Shimadzu, Kyoto, Japan) equipped with air-acetylene flame, following our previous procedures [2,36]. The spectrophotometric analysis was carried out following the parameters used in our previous research [36]. The following wavelengths (nm) were taken into account: Ca = 422.7, Cu = 342.7, Fe = 248.3, Mg = 285.2, Mn = 279.5, Zn = 213.8, K = 589.0, and Na = 766.5.

### 2.6. Total Free Amino Acid Content Determination

The sample (3.7 g flour or bread) was prepared in accordance with a method that had been previously published [37]. The filtered supernatant (100 µL, using 0.45-µm syringe filters) was determined with EZ:faast GC-MS kit (Phenomenex, Torrance, CA, USA). Amino acids were identified with a GC MS-QP 2010 Plus (Shimadzu, Kyoto, Japan) using a Zebron ZB-AAA GC column (10 m × 0.25 mm) when injection split was 1:15, carrier helium gas of 1.1 mL/min, and oven program, 30 °C/min from 110 °C to 320 °C. The MS parameters included: a source temperature of 240 °C, a scan range of 45–450 *m*/*z*, and a sampling rate of 3.5 scans/s [38].

### 2.7. Dough Rheological Test

The determination of the dough dynamic rheological characteristics was performed by using a Thermo-HAAKE, MARS 40 (Karlsruhe, Germany) with parallel plate–plate geometry (gap width of 4 mm), following Mironeasa et al.’s [39] method. The rheological behavior was evaluated by the elastic modulus (G′), viscous modulus (G″), viscosity factor (tan δ), maximum gelatinization temperature (T_max_), and creep-recovery compliance (Jc_max_, Jr_max_).

The α-amylase activity was assessed with a Falling Number equipment (FN 1305, Perten Instruments AB, Stockholm, Sweden), and the test also provided valuable information about starch grain quality [40].

The flour thermo-mechanical properties were determined by using the Mixolab device (Chopin Technology, Villeneuve La Garenne, France), through the Chopin+ protocol, following the ICC 173 method [34]. The thermo-mechanical parameters evaluated included the water absorption, WA (%), dough development time, DT (min), dough stability, ST (min), the strength of the protein network while heating the dough (C1-2), the intensity of starch gelatinization (C3-2), starch breakdown (C3-4), and starch recrystallization (C5-4).

The biaxial extension behavior of wheat/composite dough was evaluated by using a Chopin Alveograph NG-97 (Tripette et Renaud, Villeneuve-la-Garenne, France) [34]. The recorded alveogram represents dough tenacity (P), dough extensibility (L), dough strength (W), and alveographic ratio (P/L).

The gaseous release and the batter development were assessed by using the Rheofermentometer device, type F4 (Chopin Rheo, Villeneuve-La-Garenne, France) [41]. The registered characteristics of the formulated samples were the maximum height of gaseous production (H′_m_), total carbon dioxide volume production (VT), the volume of the gas retained in the dough at the end of the test (VR), and the gas retention coefficient (RC).

### 2.8. Bread Physical and Textural Characteristics Determination

The bread weight was measured with an analytical balance. Bread volume (BV) was determined by the rapeseed displacement method and specific volume (Sp_Volume), expressed as the volume/weight ratio of bread (cm^3^/g), and porosity and elasticity were calculated based on the Romanian standard [42].

The texture parameters, firmness, springiness, cohesiveness, gumminess, resilience and masticability were registered following the procedure previously presented [6]. 

The bread color was measured at five opposite points around each sample with a chromameter (CR-400, Konica Minolta, Tokyo, Japan) and expressed in the CIE*Lab* color system, where L* value indicated the lightness from black (0) to white (100), value a* ranged from green (−) to red (+), and value b* varied from blue (−) to yellow (+).

### 2.9. Sensory Evaluation of Breads

A sensory evaluation of the loaves was performed by 13 semi-trained judges 2 h after cooling. The sensory analysis methodology used in this study was approved by the Ethical Committee of the Food Engineering Department of Stefan cel Mare University [43]. The panelists were informed regarding the aim, protocols, and methodology of the study and gave their consent to participate. During the evaluation test, one slice of each type of optimal bread and the control bread were analyzed under white light in sensory test rooms with special cabins, and the judges rinsed their mouths with water between samples. The following characteristics were assessed: overall acceptability, appearance, crust and crumb structure, taste, and smell using a hedonic scale. The questionnaire used included a 9-point scale, where the score of 1 represented “dislike very much″, a score of 5, “neither like nor dislike″, and a score of 9 “extremely like″.

### 2.10. Statistical Analysis

All the experiments were conducted at least in duplicate, and the data expressed as mean ± standard deviation. Statistical analyses were carried out using IBM SPSS Statistics 25.0 (trial version, New York, NY, USA) and XLSTAT 2021.2.1 software (Addinsoft, New York, NY, USA). The one-way analysis of variance (ANOVA) was applied to assess significant differences among samples at a 95% confidence interval. The multivariate data analysis technique, principal component analysis (PCA), was used to discriminate among samples and/or characteristics, and the Pearson correlation coefficient (*r*) was determined to identify correlations between the studied parameters.

## 3. Results

### 3.1. Physico-Chemical Parameters of Wheat Flour and Optimal Composite Flours

**Figure 1:**
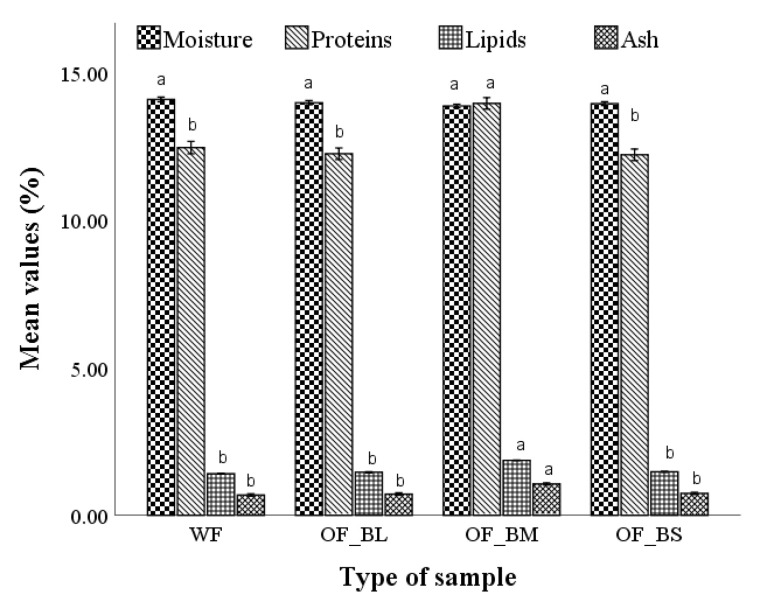
Physico-chemical parameters of wheat flour (WF) and optimal wheat–buckwheat composite flours corresponding to large (OF_BL), medium (OF_BM), and small (OF_BS) buckwheat flour particle size. All data were expressed by the mean ± standard deviation (*n* ≥ 2). Different letters (a, b) indicate significant differences (*p* < 0.05). Data are expressed on a dry basis.

Wheat flour was characterized by a slightly higher, although not significant, moisture content (14.08%), compared to optimal composite wheat–buckwheat flours which ranged from 13.86 to 13.94% (Figure 1).

The protein content of the optimal wheat–buckwheat composite flours varied between 12.00 and 14.14%, the highest percentage being present in the sample with medium PS (OF_BM) (14.14%), significantly (*p* ˂ 0.05) higher than the one with large and small PS (OF_BL and OF_ BS) and WF. The lipid content varied from 1.44 to 1.86% in optimal composite flour with large (OF_BL), medium (OF_BM), and small (OF_BM) PS. Significant differences (*p* ˂ 0.05) regarding lipid content were obtained only on the OF_BM and the other two optimal composite flour samples. The ash content of optimal composite flours varied between 0.72 and 1.64%, which is higher than that of wheat flour (0.69%). The highest ash content (1.64%) was observed in OF_BM (Figure 1). The addition of buckwheat flour at different particle sizes and distinctive doses in wheat flour clearly contributed to raising the mineral content of the optimal composite flours (Table 1). The highest contents of potassium (K) were registered in OF_BM (138.78 mg/100 g), which is remarkably higher than the other composite flour and wheat flour. Magnesium is present in higher quantities in optimal composite flours, and ranged between 156.47–157.42% compared to wheat flour (155.50%). The iron amount is statistically different in OF_BM, while OF_BL and OF_BS presented lower values, but all composite flours showed significantly higher iron values (1.99–2.55%) than wheat flour (1.80%). Regarding the flour’s sodium and manganese content, there were no significant (*p* > 0.05) differences between the samples.

**Figure 2:**
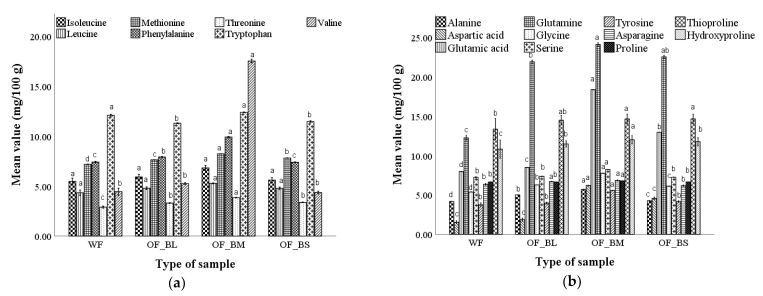
Essential (**a**) and non-essential (**b**) amino acids of wheat and optimal wheat–buckwheat composite flours with large (OF_BL), medium (OF_BM), and small (OF_BS) buckwheat particle size. Mean values (n ≥ 2) with different letters (a–d) are significantly different (*p* < 0.05). Data are expressed on a dry basis.

The investigated optimal wheat–buckwheat composite flours showed a higher amino acid content for all three fractions compared to the amino acid content characteristic of wheat flour (Figure 2a,b).

Considerably higher amounts for the following essential and non-essential amino acids compared to wheat flour were found: methionine (7.81–8.22%), threonine (3.30–3.42%), tryptophan (12.39–12.59%), valine (4.40–17.50%), alanine (4.26–5.69%), aspartic acid (1.85–6.19%), glutamic acid (8.50–18.36%), glutamine (21.88–24.09%), glycine (6.12–7.75%), serine (7.24–8.23%), tyrosine (3.97–5.55%), and asparagine (6.18–6.85%). Following the trend of nutritional composition and mineral content, also in the case of amino acid content, the highest values were observed in the OF_BM.

### 3.2. Wheat–Buckwheat Composite Flour and Bread Characterization

The application of numerical optimization in a previous study allowed the establishment of the optimal proportion of wheat:buckwheat in composite flour formulate for the large (90.87:9.13), medium (89.43:10.57), and small (89.75:10.25) buckwheat flour particle sizes, with the aim of obtaining a wheat–buckwheat composite flour and bread with the desired quality attributes (Table 2). These optimal formulations gave the best technological characteristics of wheat–buckwheat composite flour, dough, and bread as a result of a complete evaluation performed by using different devices [16]. In comparison with the control, the optimal flours presented lower values for α-amylase, dough stability, starch gelatinization, starch retrogradation, dough tenacity and extensibility, dough strength, maximum gelatinization temperature, creep-recovery compliance, and bread volume. On the other hand, dough development time, protein denaturation, stability of hot starch gel, alveographic ratio, maximum dough height during gas formation and retention, the total volume of gas formed and retained in the dough, gas retention coefficient, and bread firmness showed higher values for the optimal samples compared to the control.

The dough viscosity factor for the composite flour dough with the small particle size showed a remarkably higher value than the control, followed by the other optimal samples with large and medium particles. Overall, the optimal composite flour dough presented a decrease of creep-recovery compliance compared to the control.

### 3.3. Advanced Characterization of the Optimal Breads

#### 3.3.1. The Physical Characteristics of the Optimal Breads

Table 3 depicts the main physicochemical properties, showing the bread volume, crumb porosity, and elasticity values.

The optimal breads showed a significantly higher volume (362.51–373.19 cm^3^) than the WFB volume (352.20 cm^3^). Bread volume increased with increasing buckwheat flour particle size in composite flour, and also the bread crumb porosity and elasticity were improved. The highest values of bread volume, porosity, and elasticity were registered in OB_BL (373.19 cm^3^, 73.75%, and 94.28%, respectively).

The lightness (L*), the intensity of the red (a*) or green (−a*) and the yellow (b*) for the optimal bread crust and crumb have varied depending on distinctive dose corresponding to the particle size dimensions (Table 4).

As expected, the addition of buckwheat flour affected the color of the resulting loaves. The bread crust lightness decreased significantly in optimal breads with buckwheat flour compared to the control, with the OB_BM presenting the lowest value for this parameter. The value of the red (a*) and yellow (b*) color intensity of the control bread crust was significantly lower than that of the optimal composite flour bread samples. Bread with the optimal dose of buckwheat flour addition that was distinctive for each particle size was marked by a significantly lower lightness of the crumb, within the range of 60.71–63.28, compared to wheat bread (73.94). The value of this parameter decreased significantly with the increase of the particle size. When buckwheat flour particle size decreased, a considerable rise in redness (a*) was observed, along with a lowering of yellowness (b*) in breadcrumbs, from 17.41 to 16.15. The a* value for the optimal bread crumb varied in the negative range (from −3.91 to −2.63), results which indicate that the bread crumb became darker and less yellow with the addition of buckwheat flour.

#### 3.3.2. Optimal Bread Texture

The optimal bread texture parameters compared to WFB are presented in Table 5.

The optimal bread samples showed significant (*p* < 0.05) differences for firmness, springiness, gumminess, and masticability compared to the WFB. As illustrated in Table 5, the firmness of the optimal bread decreased substantially, from 3.78 to 3.64 N, compared to the control bread (5.71 N). Springiness presented a diminution for optimal breads from 1.1196 to 0.8115 with the reduction in buckwheat flour particle size compared to wheat flour bread springiness. The cohesiveness and resilience of the optimal bread samples did not present any significant difference. A low reduction of cohesiveness and resilience was observed for the optimal bread with the small buckwheat flour particle size. The masticability parameter registered a major decrease in optimal bread (253.19–212.33) in comparison with masticability of WFB (499.73).

#### 3.3.3. Nutritional Composition and Energy Value of Optimal Breads

The chemical composition of the optimal breads presented significant differences (*p* ˂ 0.05) both among them and compared to the WFB (Table 6).

The optimal bread obtained for each buckwheat flour fraction showed a considerably different moisture content only between the sample with a medium particle size and the other two samples with large and small particle sizes, the lowest moisture content (40.21%) being observed for OB_BM. The optimal bread samples have a raised protein content compared to the WFB. The sample OB_BM showed the highest protein content (10.00%), followed by the sample OB_BS (9.70%), and these results are correlated with the nutritional value of the buckwheat flour fractions [32]. The lipid amount from optimal bread samples increased significantly from 0.14 to 0.22% in comparison with the WFB (0.01%). The ash content of the samples showed high values in OB_BM and OB_BS, whereas the carbohydrates and energy value decreased in OB_BS.

#### 3.3.4. Amino Acid Profile of Optimal Bread Samples

**Figure 3:**
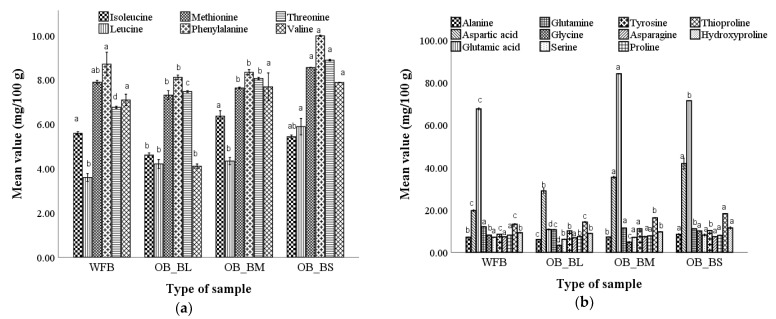
Amino acids: (**a**) essential, and (**b**) non-essential of the optimal breads. Mean values (n ≥ 2) with different letters (a–d) indicate significant differences (*p* < 0.05). Data are expressed on a dry basis.

The essential and non-essential amino acid content of the optimal bread for each buckwheat flour fraction compared to the WFB showed variations depending on the size of the fraction (Figure 3a,b).

Essential AA represents 14.60% for OB_BL, 12.20% for OB_BM, and 13.19% for OB_BS, of the total content of AA. The following variations in amino acids were registered in optimal breads: isoleucine (4.60–6.35%), leucine (4.20–5.88%), methionine (7.30–8.54%), phenylalanine (8.10–9.97%), threonine (7.46–8.87%), valine (4.40–7.87%), alanine (6.05–8.46%), aspartic acid (28.90–41.73%), glutamic acid (10.75–84.00%), glutamine (106.50–114.40%), glycine (3.25–10.05%), serine (6.00–7.65%), tyrosine (10.2–11.20%), asparagine (6.88–7.38%), proline (7.39–7.98%), thioproline (14.25–18.20%), and hydroxyproline (8.88–11.49%). An increase of essential amino acids was observed in the optimal bread compared to the WFB. Optimal breads with medium and small particle sizes were characterized by considerably higher amino acid content compared to WFB, especially leucine, methionine, threonine, valine, serine, glutamic, and aspartic acid.

#### 3.3.5. Minerals Content of Optimal Bread Samples

**Figure 4:**
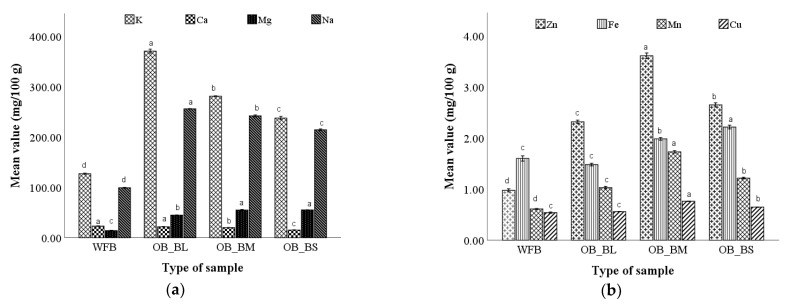
Macro- (**a**) and micro-mineral (**b**) composition of the optimal breads. Mean values (*n* ≥ 2) with different letters (a–d) are significantly different (*p* < 0.05). Data are expressed on a dry basis.

The mineral content varaition in the bread samples obtained (Figure 4) was associated with the buckwheat flour particles’ composition.

A decrease in the content of macro-elements in optimal bread was observed with the decrease of the buckwheat flour particle sizes, while the content of micro-elements showed the following increase depending on the particle size: OB_BM > OB_BS > OB_BL (Figure 4a,b). Regarding the macro-elements, the highest values were observed in OB_BL and OB_BM, while trace elements were found in OB_BM and OB_BS. It can be concluded that the richest bread in terms of mineral content is OB_BL, particularly in terms of macro-elements content.

#### 3.3.6. Sensory Evaluation of the Optimal Breads

**Figure 5:**
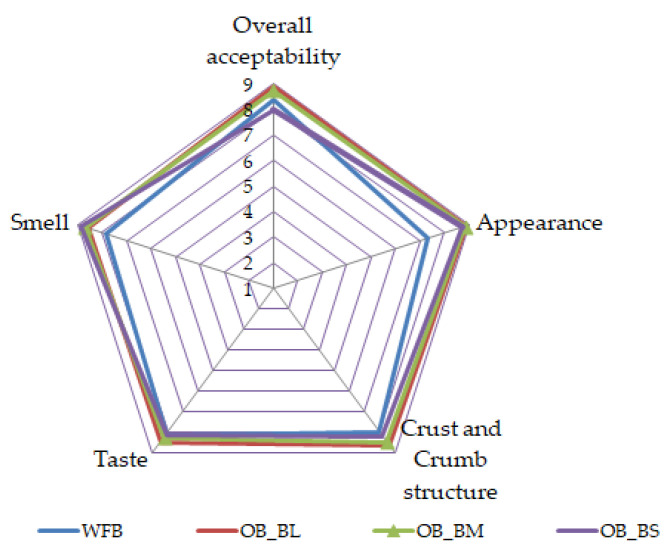
Sensory characteristics of the optimal bread samples.

The sensory evaluation results highlighted a considerable effect of the optimal buckwheat flour of large, medium, and small particle sizes which partially substituted refined wheat on the bread sensory quality and overall acceptability. The optimal bread with large and medium particle sizes (OB_BL and OB_BM) presented a higher score for sensory characteristics evaluated compared to wheat flour bread (WFB) (Figure 5).

The optimal bread made with the small particles of buckwheat flour (OB_BS) which received a general acceptability score of 8.06, compared to the score of 8.35 received by the WFB, and 8.83 and 8.76 received for the OB_BL and OB_BM, respectively. Taking into account this information, it can be concluded that all optimal breads will probably be well accepted by potential consumers.

#### 3.3.7. Evaluating Relationships between Variables

**Figure 6:**
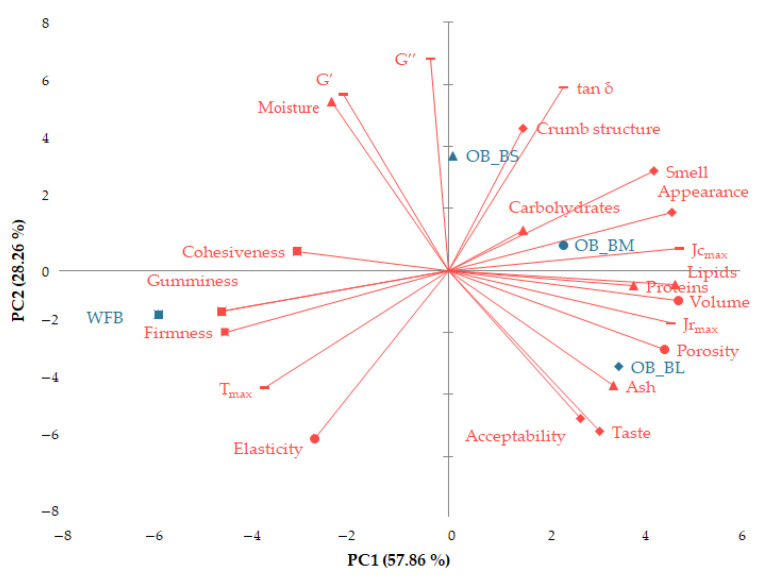
Bi-plot for the principal component analysis performed for the dough dynamic parameters (▬), physical (●), chemical (▲), textural (■), and sensory characteristics (♦) of bread. ■ WFB—Wheat flour bread; ♦ OB_BL—optimal bread with large buckwheat flour particle size; ♦ OB_BM —optimal bread with medium buckwheat flour particle size; ▲ OB_BS —optimal bread with small buckwheat flour particle size.

Principal component analysis (PCA) allowed us to visualize the similarities and/or differences between the variables. It was observed that the first two components explained 86.12% of the total variance, where the first principal component (PC1) explained 57.86% (Figure 6).

## 4. Discussion

### 4.1. Nutritional Characteristics of Optimal Wheat–Buckwheat Composition Flour

Optimal wheat–buckwheat composite flour showed proximate intermediate composition, based on the doses of buckwheat flour typical to particle sizes and to wheat flour. Similar results to those obtained in the present research regarding the protein content of optimal composite flour with particular particle sizes have been reported in some studies [13,44,45]. The results of this research confirmed that the optimal composite flour formulated is a good source of protein, lipid, and ash, which varied depending on buckwheat flour particle size. Our results are comparable to those obtained previously by other researchers [46,47,48]. Most lipids are present in the grain germ and coating, and lower quantities in the pericarp and endosperm [49,50]. Compared to wheat flour, buckwheat is rich in iron, zinc, copper, and manganese [51,52], elements that can act as cofactors of antioxidant enzymes. Buckwheat has also been reported to have a high amount of magnesium, phosphorus, and potassium, but a low amount of calcium [51]. The high ash amount of the OF_BM can be attributed to the high amount of magnesium and potassium stored in the seed embryo [53]. Supplementation of wheat flour with different fractions of buckwheat flour can increase the mineral content of formulated composite flours. The high content of potassium, phosphorus, calcium, magnesium, and iron in food may have benefits for the prevention of osteopenia and osteoporosis [54]. The results obtained for amino acid content were similar to the previous values reported by other authors who evaluated the amino acid contents in buckwheat grains and flour [55,56,57]. The amino acid content is dependent on the protein content of the otimal composite flour used in the manufacturing recipe. During the technological process, they undergo transformations, being hydrolyzed by proteolytic enzymes into amino acids and peptides with increasingly smaller molecular mass. Since all the analyzed samples were obtained under identical laboratory conditions and probably their proteolytic activity was similar, total amino acid content was dependent on the initial protein content of the optimal wheat–buckwheat composite flours used in the technological process.

### 4.2. The Technological Behavior of the Optimal Wheat–Buckwheat Composite Flour and the Bread Baking Parameters

Technological behaviour differences occurred between optimal wheat–buckwheat flour samples and the control one. This phenomenon can be explained by the non-gluten matrix, fats, minerals, and sugars from the buckwheat flour particle sizes and their interactions [32], but also some bioactive compounds present in buckwheat flour. Specialized studies have shown that the small particles of buckwheat flour are rich in phenolic compounds [58], so in the optimal composite flour containing small particles, they will form links with α-amylase, which will lead to a decline in α-amylase activity.

The reofermentometric indicators for all optimal wheat–buckwheat composite dough increased, a fact which can be explained by the fermentable carbohydrates from buckwheat flour and also by the biphasic method. Consequently, bread volume registered an increase in all samples, compared to WFB. This phenomenon may be related to the lower amount of gluten replaced, which will generate spatial structure modification of gliadin and glutenin molecular chains, with strong disulfide bonds that lead to inter- or intramolecular interactions of disulfide, ionic, or hydrogen bonds [59]. Some authors affirmed that the replacement of wheat flour with a high content of buckwheat flour (up to 20%) determined a decrease in loaves’ volume, probably due to the change in the gluten network and the fiber content which are able to retain fermentation gases [56,60].

The increase of G′ and G″ moduli was registered for all of the optimal samples compared to the WFB. This behaviour could be due to the limited plasticizing effect that favors gluten aggregation and determines a more elastic behavior of this latter sample. The highest increase in the visco-elastic moduli was registered in composite flour dough with a small particle size, variations that are probably influenced by the α-amylase amount of the optimal flour. The maximum gelatinization temperature dropped considerably compared to the control, the lower value being found for composite flour dough with a small buckwheat flour particle zise (OF_BS). The dough with a medium particle size of buckwheat flour (OF_BM) showed a viscosity factor and a maximum recovery compliance comparable to the values obtained for wheat flour dough.

### 4.3. Complex Characterization of the Optimal Bread Samples

The improved properties of the buckwheat flour-based bread were impacted by the flour’s chemical composition. This phenomenon can be related to the biphasic method of bread preparation, as well, and can be due to the hydrolysis of buckwheat proteins. Similar results regarding bread supplementation with buckwheat flour were obtained by Diowksz and Sadowska [61]. All optimal breads presented improved technological parameters compared to wheat flour bread. The best bread volume and firmness were obtained for optimal bread with medium and large buckwheat flour particle sizes. This fact can be due to the nutritional profile of optimal composite flour which was considerably improved compared to this wheat flour.

The lightness (L*) parameter plays a key role in the bakery industry. It was shown that optimal breads presented a darker crust and crumb than wheat bread which can be explained by the increased fiber content from buckwheat flour. The lowest L* value (60.71) was obtained for the sample with the large particle size, suggesting a dark bread. Our results fall in line with those obtained by Psodorov et al. [60] who observed an increase in redness and yellowness intensity, and a decrease in bread lightness with an increase in buckwheat flour dose. On the other hand, the amino acids present in optimal composite flours participate in the formation of melanoidins that color the crust of the bread.

The texture parameters of the optimal breads presented significantly lower firmness compared to the control bread which could be possibly due to the fiber and polyphenols from buckwheat flour which may cause the noncovalent interactions among diverse components. Additionally, optimal bread baking was distinguished by remarkably lower crumb springiness and gumminess compared to wheat bread. The obtained results fall in line with those reported by Kowalski et al. [56], who observed a decrease in firmness, gumminess, and chewiness when 5–50% buckwheat flour was added in wheat bread. In contrast, other authors found an increase in these textural parameters when buckwheat flour was used to substitute wheat flour [62].

The partial replacement of refined wheat flour with pseudocereal flour influenced the quality and nutritional value of the bread depending on the particle size and the optimal dose. From the point of view of the nutritional value, due to the protein quality of the pseudocereal flour given by the amino acid content, an improvement of the bakery products can be obtained when refined wheat flour is substituted with pseudocereal flour [63]. Mineral content values were generally higher in the optimal breads containing buckwheat, depending on particle size. These minerals were also found at higher levels in optimal composite flour than in the wheat flour (Table 1), and the raw material contents affect the bread’s nutritional composition. The results obtained for mineral contents in optimal bread are in agreement with those reported by Bilgiçli and İbanoğlu [64], who studied the impact of buckwheat flour on wheat bread quality.

The highest total scores for the sensory attributes were achieved for the optimal bread with large and medium buckwheat flour particle size, where the appearance, crumb structure, crust, smell, and taste had the best scores. The optimal bread with a small particle size was distinguished by the highest score for smell due to the amino acids’ contribution to flavoring substance formation. Similar results were obtained by other scholars when wheat flour was replaced with up to 20% buckwheat flour [64], but the particle size was not taken into account. These results show that bread can be successfully manufactured using optimal composite flour with buckwheat flour being up to at least a 10% addition to wheat flour, obtaining the best nutritional and texture results and a very good acceptance. At the same time, the optimal bread with a medium particle size can fulfil consumer needs from a nutritional point of view.

### 4.4. The Comprehensive Overview of Dough and Bread Characteristics

By applying the Pearson correlation analysis for the dough’s dynamic rheological parameters, chemical composition, physical and textural parameters, and sensory characteristics of the bread obtained from wheat–buckwheat composite flours, a series of correlation coefficients with values between 0.95 and 0.99 were identified. The elastic modulus (G′) of the dough samples was positively correlated with the moisture of the bread samples (r = 0.99) but negatively correlated with the bread taste (r = −0.95). Indirect relationships were obtained between bread moisture and taste (r = −0.96). The lipid content of bread samples was positively correlated with bread volume (r = 0.95), but negatively correlated with bread gumminess and chewiness (r = −0.96). The ash content of the bread was positively associated with bread acceptability (r = 0.97). PC1 was associated with the maximum gelatinization temperature, the maximum creep and recovery compliance of the dough, and the content of proteins, lipids, ash, firmness, gumminess, volume, appearance, and smell of breads. It was observed that PC1 highlighted the opposition between the control bread and the OB_BM. The OB_BL showed a higher ash content, maximum recovery compliance, volume, porosity, taste, and acceptability, while the wheat bread showed high values for texture parameters and maximum gelatinization temperature. PC2 was associated with the elastic and viscous moduli, the viscosity factor (tan δ), and the moisture of the bread samples and crumb structure, and the highest values of these variables were obtained for the OB_BS.

## 5. Conclusions

This work complements the previous study through complete characterization of the optimal wheat–buckwheat composite flours with large, medium, and small particle sizes established in there. Additionally, valuable information regarding the bread features was determined. The results showed an increase in volume, porosity, and elasticity for all loaves, whereas the firmness, crust, and crumb lightness decreased, compared to wheat bread. This trend is associated with the technological characteristics of optimal composite flours. Among all the optimal bread samples that with the medium particle (OB_BM) presents the highest protein and lipid content and had the best nutritional profile. Additionally, an improvement in amino acid content was obtained, but the highest values were registered for bread with small (OB_BS) and medium (OB_BM) particle sizes. Regarding the mineral profile of the bread samples, the highest content was obtained for that with large particle size (OB_BL), especially in terms of macro-element contents. The sensory evaluation highlighted that the panelists appreciated the breads made from optimal composite flours with large (OB_BL) and medium (OB_BM) particle sizes the most. These findings advocate for the use of wheat–buckwheat composite flour with large, medium, and small particle sizes to enhance the physical, textural, nutritional, and sensorial features of bread.

## Figures and Tables

**Table 1:** Mineral content of wheat and optimal wheat–buckwheat composite flours.

Elements (mg/100 g)	WF	OF_BL	OF_BM	OF_BS
K	108.50 ± 2.12 ^b^	108.50 ± 2.12 ^b^	139.78 ± 34.64 ^a^	108.05 ± 10.59 ^b^
Ca	24.80 ± 0.10 ^a^	24.20 ± 0.19 ^b^	23.73 ± 0.02 ^c^	24.13 ± 0.01 ^b^
Mg	155.50 ± 0.65 ^a^	156.66 ± 0.42 ^ab^	157.42 ± 0.07 ^ab^	156.47 ± 0.03 ^ab^
Na	7.33 ± 0.11 ^a^	7.60 ± 0.95 ^a^	7.72 ± 1.10 ^a^	7.62 ± 0.18 ^a^
Fe	1.80 ± 0.06 ^b^	2.22 ± 0.56 ^ab^	2.55 ± 0.05 ^a^	1.99 ± 0.01 ^ab^
Zn	3.02 ± 0.25 ^a^	3.46 ± 0.07 ^ab^	3.58 ± 0.07 ^ab^	3.20 ± 0.19 ^a^
Mn	1.59 ± 0.10 ^a^	1.98 ± 0.41 ^a^	2.25 ± 0.52 ^a^	1.64 ± 0.02 ^a^
Cu	0.56 ± 0.01 ^b^	0.65 ± 0.21 ^ab^	0.68 ± 0.07 ^a^	0.60 ± 0.01 ^ab^

WF—Wheat flour; OF_BL—optimal composite flour with large buckwheat particle size; OF_BM—optimal composite flour with medium buckwheat particle size; OF_BS—optimal composite flour with small buckwheat particle size. Mean values (n ≥ 2) on the same row followed by different letters (a–c) are significantly different (*p* < 0.05). Data are expressed on a dry basis.

**Table 2:** Optimal wheat–buckwheat composite flour, dough rheology, and bread characteristics.

Characteristic	WF	OF_BL	OF_BM	OF_BS
FN (s) **Mixolab**	312.00 ± 5.25 ^axA^	317.22 ± 5.25 ^b^	316.52 ± 8.15 ^y^	352.01 ± 9.38 ^B^
WA (%)	58.50 ± 0.02 ^axA^	59.50 ± 0.25 ^a^	58.55 ± 0.50 ^x^	59.20 ± 0.17 ^A^
DT (min)	1.69 ± 0.75 ^axB^	2.85 ± 0.55 ^b^	2.95 ± 0.50 ^y^	1.45 ± 0.80 ^A^
ST (min)	9.96 ± 0.65 ^byB^	9.25 ± 0.66 ^a^	9.50 ± 2.15 ^x^	8.90 ± 0.96 ^A^
C1-2 (N·m)	0.61 ± 0.02 ^axA^	0.63 ± 0.25 ^a^	0.64 ± 0.02 ^x^	0.65 ± 0.03 ^A^
C3-2 (N·m)	1.41 ± 0.03 ^axB^	1.35 ± 0.03 ^a^	1.40 ± 0.01 ^x^	1.42 ± 0.0 ^A^
C3-4 (N·m)	0.05 ± 0.04 ^axA^	0.12 ± 0.02 ^b^	0.11 ± 0.01 ^y^	0.05 ± 0.03 ^A^
C5-4 (N·m) **Alveograph**	1.15 ± 0.01 ^byB^	0.95 ± 0.01 ^a^	0.92 ± 2.01 ^x^	0.89 ± 0.07 ^A^
P (mm H_2_O)	87.00 ± 5.75 ^byB^	80.52 ± 10.15 ^a^	82.05 ± 3.57 ^x^	84.23 ± 4.57 ^A^
L (mm)	91.00 ± 10.50 ^byB^	51.55 ± 9.50 ^a^	52.50 ± 8.52 ^x^	48.81 ± 12.42 ^A^
W (10^−4^ J)	253.00 ± 20.14 ^byB^	130.58 ± 15.10 ^a^	127.58 ± 5.58 ^x^	138.01 ± 18.22 ^A^
P/L (adim.) **Rheofermentometer**	0.95 ± 0.05 ^axA^	1.47 ± 0.50 ^b^	0.97 ± 0.52 ^x^	1.79 ± 0.49 ^A^
H′_m_ (mm)	62.00 ± 4.25 ^axA^	75.25 ± 2.25 ^b^	71.25 ± 6.23 ^y^	70.75 ± 2.50 ^B^
VT (mL)	1168.00 ± 89.56 ^axA^	1345.05 ± 85.25 ^b^	1295.45 ± 120.40 ^y^	1346.88 ± 100.58 ^B^
VR (mL)	991.20 ± 85.25 ^axA^	1200.45 ± 85.25 ^b^	1128.50 ± 595.70 ^y^	1221.78 ± 64.25 ^B^
CR (%) **Rheometer**	84.20 ± 2.50 ^axA^	91.45 ± 1.45 ^b^	90.85 ± 4.75 ^y^	91.94 ± 5.58 ^B^
G′ (Pa)	26,370.00 ± 10.00 ^axA^	26,952.25 ± 214.78 ^a^	26,950.15 ± 265.45 ^y^	27,087.10 ± 1763.00 ^B^
G″ (Pa)	9488.00 ± 74.58 ^bxA^	9647.25 ± 685.45 ^b^	9850.45 ± 456.52 ^y^	10,636.45 ± 681.85 ^B^
tan δ (adim.)	0.3610 ± 0.02 ^bxB^	0.3580 ± 0.01 ^a^	0.3600 ± 0.05 ^x^	0.3801 ± 0.01 ^B^
T_max_ (°C)	83.24 ± 0.55 ^byB^	80.25 ± 1.00 ^a^	81.25 ± 0.52 ^x^	79.42 ± 0.91 ^A^
Jc_max_ (10^−5^ Pa^−1^)	24.50 ± 4.50 ^bxB^	23.25 ± 2.25 ^a^	22.80 ± 1.50 ^x^	22.56 ± 2.75 ^A^
Jr_max_ (10^−5^ Pa^−1^)	16.62 ± 2.40 ^bxA^	15.55 ± 1.50 ^a^	16.75 ± 1.50 ^x^	15.66 ± 5.25 ^A^

WF—wheat flour; OF_BL—optimal composite flour with large buckwheat particle size; OF_BM—optimal composite flour with medium buckwheat particle size; OF_BS—optimal composite flour with small buckwheat particle size. Mean values on the same row followed by different letters are significantly different (*p* < 0.05): a, b (OF_BL), x, y (OF_BM), and A, B (OF_BS), respectively, for differences between the control and optimal formulations values. FN—Falling Number; WA—water absorption capacity; DT—development time; ST—dough stability; C1-2—protein denaturation; C3-2—starch gelatinization; C3-4—stability of hot starch gel; C5-4—starch retrogradation; P—tenacity; L—extensibility; W—deformation energy; P/L—alveographic ratio; H′_m_—maximum height; VT—total volume of gas; VR—volume of gas retained; CR—gas retention coefficient; G′—elastic modulus; G″—viscous modulus; tan δ—viscosity factor; T_max_—maximum gelatinization temperature; Jc_max_—maximum creep compliance; Jr_max_—maximum recovery compliance.

**Table 3:** Physical parameters of the optimal breads.

Bread Sample	Bread Volume (cm^3^)	Specific Volume (cm^3^/g)	Porosity (%)	Elasticity (%)
WFB	352.20 ± 15.25 ^d^	2.45 ± 0.25 ^c^	64.22 ± 5.62 ^c^	91.70 ± 6.52 ^d^
OB_BL	373.19 ± 22.25 ^a^	2.76 ± 0.35 ^a^	73.75 ± 5.85 ^a^	94.28 ± 8.45 ^a^
OB_BM	370.25 ± 25.45 ^b^	2.70 ± 0.10 ^b^	72.79 ± 4.78 ^b^	93.58 ± 5.52 ^b^
OB_BS	362.51 ± 58.70 ^c^	2.55 ± 0.52 ^b^	72.57 ± 2.75 ^b^	92.41 ± 4.78 ^c^

WFB—Wheat flour bread; OB_BL—optimal bread with large buckwheat flour particle size; OB_BM—optimal bread with medium buckwheat flour particle size; OB_BS—optimal bread with small size buckwheat flour particle. Mean values (*n* ≥ 2) on the same column followed by different letters are significantly different (*p* < 0.05).

**Table 4:** Color parameters of the optimal breads.

Bread Sample	Crust Color Parameters			Crumb Color Parameters		
	L*	a*	b*	L*	a*	b*
WFB	70.35 ± 0.91 ^a^	−1.33 ± 0.22 ^d^	32.27 ± 0.28 ^c^	73.94 ± 0.27 ^a^	−4.48 ± 0.03 ^b^	20.02 ± 0.23 ^a^
OB_BL	63.25 ± 0.99 ^b^	3.83 ± 0.55 ^c^	33.82 ± 0.37 ^bc^	60.71 ± 0.48 ^b^	−3.20 ± 0.11 ^a^	16.15 ± 0.02 ^c^
OB_BM	57.47 ± 0.83 ^c^	6.59 ± 0.43 ^a^	35.24 ± 0.83 ^ab^	61.63 ± 2.12 ^b^	−2.63 ± 0.11 ^a^	17.41 ± 0.28 ^b^
OB_BS	63.92 ± 0.67 ^b^	4.88 ± 0.03 ^b^	37.40 ± 0.90 ^a^	63.28 ± 0.48 ^b^	−3.91 ± 0.01 ^b^	16.16 ± 0.29 ^c^

WFB—Wheat flour bread; OB_BL—optimal bread with large buckwheat flour particle size; OB_BM—optimal bread with medium buckwheat flour particle size; OB_BS—optimal bread with small buckwheat flour particle size. L*—lightness of samples; a*—redness or greenness of samples; b*—yellowness of sample. Mean values (*n* ≥ 2) on the same column followed by different letters are significantly different (*p* < 0.05).

**Table 5:** Texture parameters of the optimal breads.

Bread Sample	Firmness (N)	Springiness (Adim.)	Cohesiveness (Adim.)	Gumminess (N)	Resilience (Adim.)	Masticability (N)
WFB	5.71 ± 0.02 ^a^	1.3457 ± 0.27 ^a^	0.8575 ± 0.01 ^a^	499.73 ± 4.63 ^a^	1.8278 ± 0.00 ^a^	499.73 ± 4.63 ^a^
OB_BL	3.68 ± 0.06 ^b^	1.1196 ± 0.00 ^b^	0.8492 ± 0.03 ^a^	212.33 ± 17.70 ^c^	1.9335 ± 0.17 ^a^	212.33 ± 17.70 ^b^
OB_BM	3.64 ± 0.26 ^b^	1.0157 ± 0.00 ^bc^	0.8376 ± 0.03 ^a^	221.58 ± 12.54 ^c^	1.9323 ± 0.07 ^a^	221.58 ± 12.54 ^b^
OB_BS	3.78 ± 0.02 ^b^	0.8115 ± 0.00 ^c^	0.8570 ± 0.00 ^a^	253.19 ± 13.00 ^b^	1.8114 ± 0.06 ^a^	253.19 ± 13.00 ^b^

WFB—Wheat flour bread; OB_BL—optimal bread with large buckwheat flour particle size; OB_BM—optimal bread with medium buckwheat flour particle size; OB_BS—optimal bread with small buckwheat flour particle size. Mean values (*n* ≥ 2) on the same column followed by different letters are significantly different (*p* < 0.05).

**Table 6:** Physico-chemical properties of optimal breads.

Bread Sample	Moisture (%)	Proteins (%)	Lipids (%)	Ash (%)	Carbohydrates (%)	Energetic Value (kcal)
WFB	43.12 ± 0.03 ^b^	8.35 ± 0.13 ^c^	0.01 ± 0.00 ^c^	0.72 ± 0.02 ^b^	47.81 ± 0.11 ^a^	230.31 ± 0.22 ^a^
OB_BL	43.23 ± 0.03 ^a^	9.31 ± 0.25 ^b^	0.15 ± 0.02 ^a^	0.85 ± 0.02 ^a^	46.46 ± 0.18 ^b^	230.05 ± 0.12 ^a^
OB_BM	40.21 ± 0.07 ^c^	10.00 ± 0.14 ^a^	0.22 ± 0.02 ^a^	0.85 ± 0.01 ^a^	48.71 ± 0.10 ^a^	242.75 ± 0.15 ^ab^
OB_BS	43.64 ± 0.19 ^a^	9.70 ± 0.13 ^b^	0.14 ± 0.02 ^b^	0.69 ± 0.03 ^b^	45.84 ± 0.06 ^a^	229.01 ± 1.00 ^bc^

WFB—Wheat flour bread; OB_BL—optimal bread with large buckwheat flour particle size; OB_BM—optimal bread with medium buckwheat flour particle size; OB_BS—optimal bread with small buckwheat flour particle size. Mean values (*n* ≥ 2) on the same column followed by different letters are significantly different (*p* < 0.05). Data are expressed on a dry basis.

## Data Availability

Data is contained within the article.
